# Orientation Engineering in Flexible Ag_2_Se‐Based Thermoelectric Films

**DOI:** 10.1002/advs.76925

**Published:** 2026-07-30

**Authors:** Hao Wu, Xiao‐Lei Shi, Qingfeng Liu, Zhi‐Gang Chen

**Affiliations:** ^1^ State Key Laboratory of Materials‐Oriented Chemical Engineering College of Chemical Engineering Nanjing Tech University Nanjing China; ^2^ School of Chemistry and Physics ARC Research Hub in Zero‐emission Power Generation for Carbon Neutrality, and Centre for Materials Science Queensland University of Technology Brisbane Queensland Australia

**Keywords:** biocompatibility, materials science, nanotechnology, nanowire, power factor, thermoelectric effect, thermoelectric materials, wearable computer, wearable technology

## Abstract

Flexible thermoelectrics convert body heat into electricity, offering a promising route towards self‐powered wearable electronics while overcoming the limitations of conventional batteries. Among emerging flexible thermoelectric materials, silver selenide (Ag_2_Se) has attracted widespread attention because it combines outstanding near‐room‐temperature thermoelectric performance with low cost, excellent mechanical flexibility, and superior biocompatibility. Over the past five years, orientation engineering has emerged as an effective strategy for simultaneously enhancing carrier transport and suppressing carrier scattering, leading to remarkable improvements in both material properties and device performance. In this Perspective, we systematically review recent progress in highly oriented Ag_2_Se films, including deposited, nanowire‐based, selenized, and free‐standing architectures. We further propose film thickness together with near‐room‐temperature power factor as practical metrics for benchmarking their application potential. By correlating fabrication strategies, microstructural evolution, crystallographic orientation, and thermoelectric performance, we establish a unified framework for understanding orientation‐dependent charge transport in Ag_2_Se films. Finally, we discuss the remaining scientific and technological challenges and highlight future opportunities for developing scalable, mechanically robust, and high‐performance Ag_2_Se films for next‐generation wearable thermoelectric energy harvesters and self‐powered physiological monitoring systems.

## Graphical abstract

We comprehensively review recent advances in highly oriented Ag_2_Se films. We propose two practical metrics to quantitatively benchmark their application potential and establish a unified framework that elucidates the orientation‐dependent charge transport mechanism in Ag_2_Se‐based films. Finally, we identify the remaining scientific challenges and outline future directions for the development of high‐performance Ag_2_Se films.

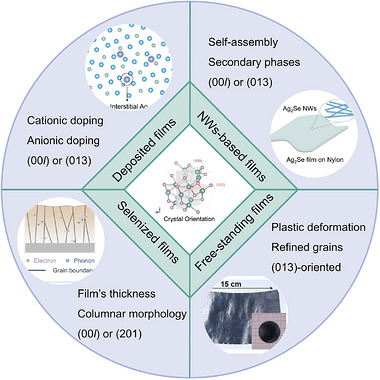

## 1 Introduction

Flexible electronics, fabricated from organic, inorganic, or composite materials, enable the development of bendable and foldable devices for wearable and biomedical applications [1, 2]. However, the operational lifetime of such systems is often constrained by the rigidity and limited capacity of conventional batteries. Flexible thermoelectric devices (F‐TEDs) can harvest heat from the human body and directly convert it into electricity, thereby providing a promising route toward self‐powered systems [3, 4, 5]. The energy conversion efficiency of F‐TEDs is largely determined by the dimensionless figure of merit (*ZT*) of thermoelectric materials near room temperature [6, 7], which is defined as *ZT* = *S* ^2^ *σT*/*κ*. Here, *S* ^2^ *σ* represents the power factor, where *S* is the Seebeck coefficient, *σ* is the electrical conductivity, *T* denotes the absolute temperature, and *κ* is the thermal conductivity. The *κ* can be further decomposed into the lattice thermal conductivity (*κ*_l_) and the electronic thermal conductivity (*κ*_e_) [8, 9]. Classical thermoelectric materials, such as Bi_2_(Te, Se)_3_, (Bi, Sb)_2_Te_3_, and Ag_2_Se, are promising candidates for the fabrication of F‐TEDs owing to their high *S* ^2^ *σ* and *ZT* values near room temperature [10, 11, 12, 13, 14, 15]. For instance, the carbon nanotube (CNT)‐supported Bi_2_Te_3_ film delivers an exceptional room‐temperature *S* ^2^ *σ* of ∼16 µW cm^−1^ K^−2^ and a *ZT* of ∼0.89, while the Te‐incorporated Ag_2_Se film exhibits a high *S* ^2^ *σ* of 25.7 µW cm^−1^ K^−2^ at 303 K, leading to an admirable *ZT* value of 1.06 near room temperature. However, Te‐based materials suffer from issues related to toxicity, elemental scarcity, and intrinsic brittleness, which significantly restrict their application scenarios, particularly in wearable thermoelectric systems. In contrast, Ag_2_Se‐based materials are more environmentally benign and cost‐effective [16]. Moreover, rational alloying strategies endow Ag_2_Se with excellent room‐temperature plasticity, making it a highly promising candidate for F‐TEDs [17, 18].

**FIGURE 1:**
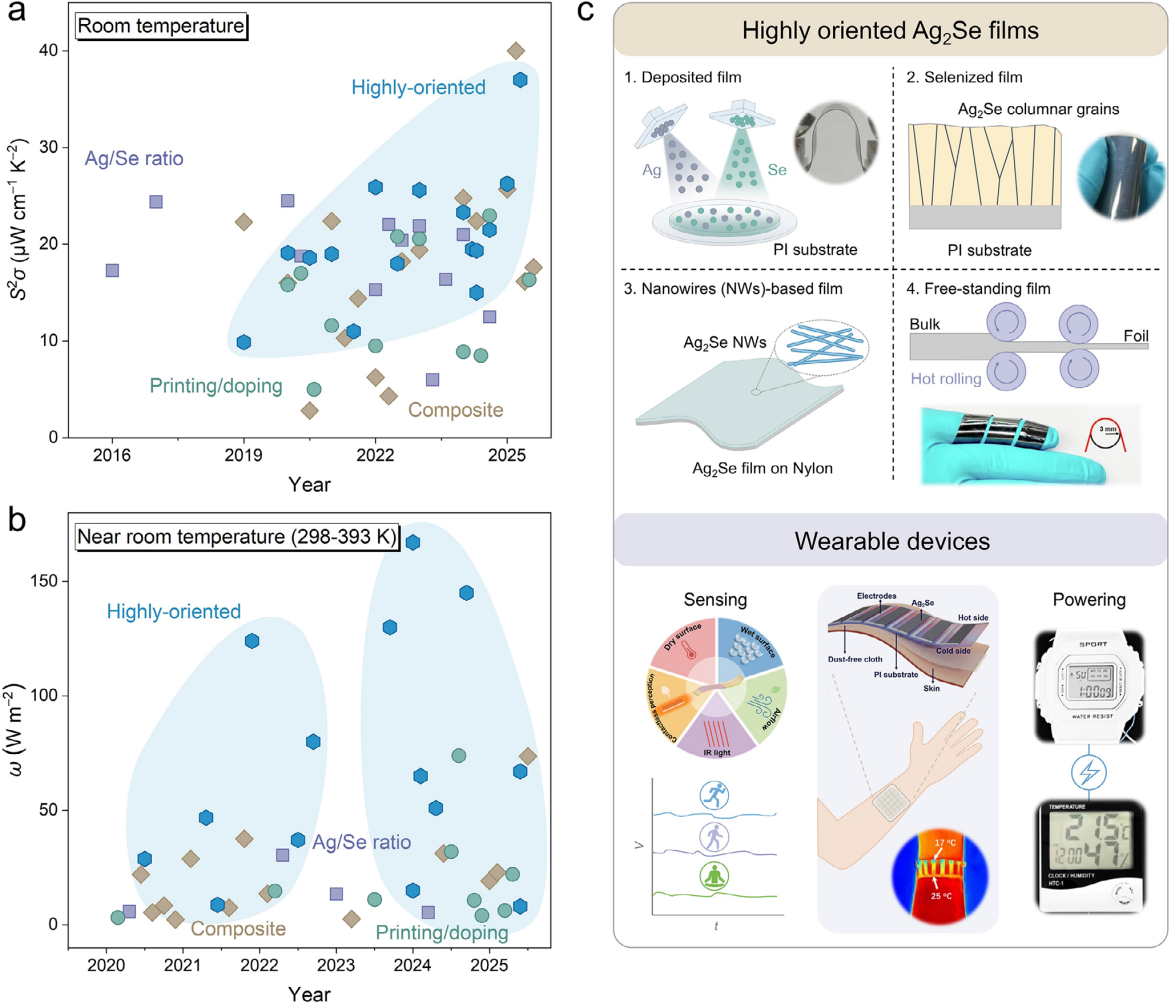
Overview of highly oriented Ag_2_Se films and their devices. (a) Room‐temperature power factor (*S* ^2^ *σ*) of flexible Ag_2_Se‐based thermoelectric films [13, 14, 20, 21, 23, 25, 26, 27, 28, 29, 30, 31, 32, 33, 34, 35, 37, 38, 39, 40, 42, 43, 44, 45, 46, 47, 48, 49, 50, 51, 52, 53, 54, 55, 56, 57, 58, 59, 60, 61, 62, 63, 64, 65, 66, 67, 68, 69, 70, 71, 72, 73, 74, 75, 76, 77]. (b) Maximum power density (*ω*) of Ag_2_Se‐based devices near room temperature [13, 20, 21, 22, 23, 24, 25, 26, 28, 29, 30, 31, 33, 34, 35, 38, 39, 40, 48, 54, 55, 58, 60, 61, 62, 64, 65, 66, 67, 68, 69, 71, 72, 73, 74, 75, 76, 77]. (c) Classifications of highly oriented Ag_2_Se films and their representative application scenarios. Here, polyimide is abbreviated as PI; infrared is abbreviated as IR. Highly oriented Ag_2_Se films (deposited film: reproduced with permission [73]. Copyright 2024, Springer Nature; selenized film: reproduced with permission [69]. Copyright 2022, Wiley; free‐standing film: reproduced with permission [74]. Copyright 2024, Springer Nature). Wearable devices (sensing: reproduced with permission [59]. Copyright 2024, American Chemical Society; powering: reproduced with permission [77]. Copyright 2025, Springer Nature; middle upper: reproduced with permission [54]. Copyright 2022, Elsevier; middle lower: reproduced with permission [74]. Copyright 2024, Springer Nature).

Ag_2_Se is a typical narrow‐band‐gap semiconductor that exhibits intrinsically high carrier mobility (*μ*) and low *κ*_l_ near room temperature [19]. To improve the thermoelectric performance and flexibility of Ag_2_Se‐based films, several strategies have been developed, including composite engineering (such as hybridization with organic components or incorporation of inorganic phases) [13, 20, 21, 22, 23, 24, 25, 26], stoichiometric ratio modulation [27, 28, 29, 30], and doping [31, 32, 33, 34]. Besides, printing techniques are widely utilized to realize large‐scale fabrication of Ag_2_Se‐based films [35, 36, 37, 38, 39, 40, 41]. As a result, some flexible Ag_2_Se‐based films have achieved *S* ^2^ *σ* exceeding 20 µW cm^−1^ K^−2^ at room temperature (Figure 1a) [13, 14, 20, 21, 23, 25, 26, 27, 28, 29, 30, 31, 32, 33, 34, 35, 37, 38, 39, 40, 42, 43, 44, 45, 46, 47, 48, 49, 50, 51, 52, 53, 54, 55, 56, 57, 58, 59, 60, 61, 62, 63, 64, 65, 66, 67, 68, 69, 70, 71, 72, 73, 74, 75, 76, 77]. Recently, strongly anisotropic electrical and thermal transport behaviors have been observed in both single‐crystal and polycrystalline Ag_2_Se [78, 79]. Given the well‐established consensus that orientation engineering can effectively optimize the thermoelectric performance of flexible Bi_2_Te_3_‐based films [10, 11, 80], research efforts have increasingly shifted toward applying orientation engineering to Ag_2_Se‐based films. Consequently, highly oriented Ag_2_Se films have experienced rapid development over the past five years, and their room‐temperature thermoelectric performance has been greatly improved, as shown in Table 1 [20, 65, 66, 67, 68, 69, 71, 72, 73, 74, 75, 76, 77]. For example, doping‐induced orientation engineering has been proposed to produce highly (00*l*)‐oriented Ag_2_Se films with excellent structural uniformity, achieving a *ZT* of 1.27 at 363 K, comparable to that of bulk counterparts [73]. Meanwhile, a solution‐based selenization route has been developed to fabricate Ag_2_Se films with a columnar microstructure [69]. The resulting strong out‐of‐plane orientation facilitates the decoupling of electron and phonon transport, leading to an ultrahigh *ZT* of 1.2 at room temperature [69]. In addition, a plastic deformation strategy has been reported for the first time to fabricate free‐standing Ag_2_Se films with preferred crystallographic orientation, which exhibit excellent flexibility even at thicknesses exceeding 100 µm [74]. Notably, F‐TEDs fabricated from these oriented films demonstrate superior output power density (*ω*) compared with other Ag_2_Se‐based films, highlighting their strong potential for self‐powered wearable electronics (Figure 1b) [13, 20, 21, 22, 23, 24, 25, 26, 28, 29, 30, 31, 33, 34, 35, 38, 39, 40, 48, 54, 55, 58, 60, 61, 62, 64, 65, 66, 67, 68, 69, 71, 72, 73, 74, 75, 76, 77].

**TABLE 1:** A summary of the room‐temperature thermoelectric performance of the highly‐oriented Ag_2_Se films (Here, polyvinylpyrrolidone is abbreviated as PVP; reduced graphene oxide as rGO).

Product	*ZT*	*σ* (S cm^−1^)	*S* (µV K^−1^)	*S* ^2^ *σ* (µW cm^−1^ K^−2^)	*κ* (W m^−1^ K^−1^)	*n* (cm^−3^)	*μ* (cm^2^ V^−1^ s^−1^)	Year	References
Ag_2_Se	0.6	497	−141	9.87	/	3.8 × 10^18^	850	2019	[14]
Ag_2_Se + Ag	/	3958	67.5	∼18.6	/	1.6 × 10^20^	∼83	2020	[65]
Ag_2_Se + PVP	1.1	925	−144	19.1	/	7 × 10^18^	1100	2020	[20]
Ag_2_Se	/	∼730	−123	11	/	4.8 × 10^19^	94	2021	[66]
Ag_1.8_Se	/	1000	−138	19	/	7.5 × 10^18^	950	2021	[67]
Ag_2.02_Se	∼0.55	1038	−135	∼18	∼0.95	2.2 × 10^19^	∼315	2022	[68]
Ag_2_Se	∼1.2	∼1440	−135	∼25.9	∼0.66	6.5 × 10^18^	1250	2022	[69]
Cu‐doped Ag_2_Se	/	1350	−125	20.8	/	8.9 × 10^18^	∼950	2023	[70]
Ag_2.19_Se	/	∼2161	−104	23.3	/	∼5 × 10^19^	∼255	2024	[71]
S‐doped Ag_2_Se	/	∼870	−149	19.35	/	4.5 × 10^18^	1150	2024	[72]
Te‐doped Ag_2_Se	1.15	990	−146	21.1	0.55	1.3 × 10^19^	445	2024	[73]
Elastic Ag_2_Se	/	∼1360	−105	15	/	6 × 10^18^	∼1500	2024	[74]
Elastic Ag_2_Se_0.9_S_0.1_	/	∼2000	−95	∼18	/	∼1 × 10^19^	∼1200	2024	[75]
Hot rolled Ag_2_Se		1300	−125	21.4	/	7.6 × 10^18^	1089	2025	[81]
Ag_2_Se	0.9	1200	−145	∼26	∼0.88	5 × 10^18^	1440	2025	[76]
Ag_2_Se + rGO	1.28	1480	−158	37	<0.9	5.5 × 10^18^	1500	2025	[77]

According to their fabrication processes, reported highly oriented Ag_2_Se films can be broadly categorized into deposited films, selenized films, nanowires (NWs)‐based films, and free‐standing films. Each type exhibits distinct preferred crystallographic orientations and microstructural characteristics, leading to varied electrical and thermal transport properties. These tailored features enable their applications in wearable technologies such as energy harvesting and physiological sensing, as illustrated in Figure 1c [54, 59, 69, 73, 74, 77]. Given the rapid progress achieved in highly oriented Ag_2_Se films, it is timely to present a concise perspective that summarizes the key advances, identifies remaining challenges, and outlines future research directions. In this work, we first classify oriented Ag_2_Se films according to their fabrication processes and propose two quantitative indicators (film thickness and near‐room‐temperature *S* ^2^ *σ*) to compare their characteristics and application potential. We then focus on the advanced orientation engineering strategies employed in different types of oriented films, including modulation of elemental compositions and phase components, microstructural design, and plastic processing. Finally, we discuss existing controversies and challenges and provide perspectives on the future development of highly oriented Ag_2_Se films.

## 2 Classifications of Highly Oriented Ag_2_Se Films

**FIGURE 2:**
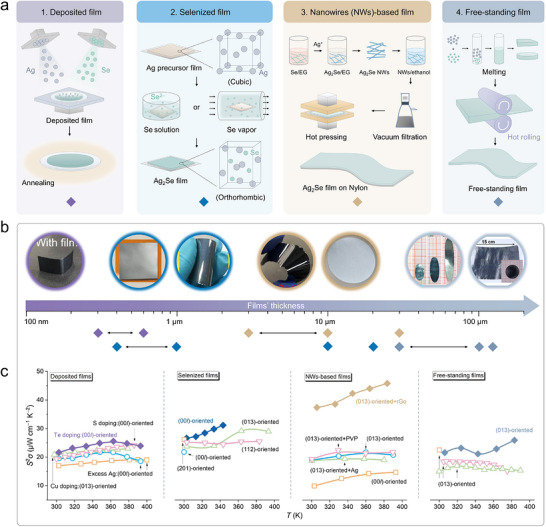
Classification and performance comparison of highly oriented Ag_2_Se films. (a) Schematic illustration of the fabrication processes for different types of oriented films, including deposited films, selenized films, NWs‐based films, and free‐standing films. Here, ethylene glycol is abbreviated as EG. (b) Representative highly oriented Ag_2_Se films across different thickness scales. Deposited film: reproduced with permission [54]. Copyright 2022, Elsevier. Selenized films (left: reproduced with permission [91]. Copyright 2025, American Chemical Society; right: reproduced with permission [69]. Copyright 2022, Wiley). NWs‐based films (left: reproduced with permission [93]. Copyright 2021, AIP Publishing; right: reproduced with permission [77]. Copyright 2025, Springer Nature). Free‐standing films (left: reproduced with permission [75]. Copyright 2024, American Chemical Society; right: reproduced with permission [86]. Copyright 2025, Springer Nature). (c) Summary of the near‐room‐temperature *S* ^2^ *σ* of highly oriented Ag_2_Se films.

Based on the fabrication methods, highly oriented Ag_2_Se films can be broadly classified into four categories: deposited films, selenized films, NWs‐based films, and free‐standing films, as summarized in Figure 2a. Deposited films are typically fabricated using vacuum thermal evaporation (VTE) [82] and magnetron sputtering (MS) [83] techniques. These approaches enable the rapid preparation of high‐quality films within minutes while allowing precise control over the Ag/Se stoichiometric ratio through adjustment of the target composition [67, 68]. Furthermore, extrinsic doping with elements such as Cu, S, or Te can be readily introduced during the deposition process [54, 73]. Owing to these advantages, deposited films provide an ideal platform for systematically investigating the effects of both Ag/Se stoichiometry and extrinsic dopants on the crystallographic orientation of Ag_2_Se films.

Selenized films represent a recently developed class of oriented Ag_2_Se films that has emerged over the past three years, whose crystallographic orientation is strongly influenced by the film thickness and selenization conditions [69, 76, 84]. The fabrication process generally involves two steps: preparation of an Ag‐containing precursor film followed by a selenization treatment, as illustrated in Figure 2a. Modulating the thickness of the Ag‐containing precursor film can effectively tune the crystal orientation of the resulting selenized Ag_2_Se films. Specifically, a thicker precursor film typically promotes a higher degree of selenization, leading to a pronounced out‐of‐plane orientation [69]. In addition, adjusting the Se concentration in Se‐containing aqueous solutions or vapor environments provides another effective route to regulate the preferred orientation of the selenized films [76].

Ag_2_Se NWs, characterized by their high aspect ratio and facile synthesis under ambient conditions, have been widely employed to fabricate flexible Ag_2_Se‐based thermoelectric films [14]. The typical fabrication process involves the synthesis of Ag_2_Se NWs, vacuum filtration of the Ag_2_Se NWs onto a nylon substrate, and subsequent hot pressing, as illustrated in Figure 2a. Recent studies have shown that the preferred crystallographic orientations and microstructures of such NWs‐based films can be effectively tuned by adjusting the NWs synthesis conditions or by adopting composite engineering strategies [20, 43, 65, 77]. Specifically, modulating the synthesis conditions to control the morphology and distribution of Ag_2_Se NWs can effectively alter the preferred orientation of the resulting films. In addition, incorporating low‐dimensional secondary phases during the NW synthesis can influence grain growth behavior, thereby further regulating the preferred orientation and microstructural characteristics of the films.

The three types of highly oriented Ag_2_Se films discussed above are not intrinsically flexible. Their bendability primarily arises from the flexibility of the supporting substrates and the enhanced elastic compliance achieved by reducing the film thickness [85]. Consequently, the output performance of devices based on these films is inherently limited once the thermoelectric performance of the films is fixed. In contrast, recent studies have demonstrated that bulk Ag_2_Se can undergo plastic deformation at moderately elevated temperatures to produce thin, free‐standing films [74, 75, 81, 86]. During this rolling process, grain elongation is induced, resulting in a pronounced (013) preferred orientation and anisotropic thermoelectric properties [81]. More importantly, the plastic deformation process endows these free‐standing films with intrinsically improved flexibility.

To better summarize and compare the characteristics of these films, two quantitative indicators (film thickness and near‐room‐temperature *S* ^2^ *σ*) are proposed from an application‐oriented perspective [87]. Since Ag_2_Se‐based F‐TEDs typically adopt lateral π‐type configurations, in which the thermoelectric legs are composed of in‐plane distributed films, the flexibility and output power of F‐TEDs are strongly correlated with the thickness of the Ag_2_Se films [16, 88]. Therefore, film thickness is employed as a key quantitative indicator to compare these highly oriented Ag_2_Se films, as illustrated in Figure 2b. Among the four types of oriented films, deposited films generally exhibit the smallest thickness, typically hundreds of nanometers, whereas selenized films possess thicknesses ranging from hundreds of nanometers to tens of micrometers. NWs‐based films can maintain excellent flexibility even at thicknesses of tens of micrometers, which can be attributed to the intrinsic bendability of Ag_2_Se NWs and the strong interfacial bonding between the film and the nylon substrate [14]. In contrast, free‐standing films typically exhibit the largest thickness, thereby offering considerable potential for practical applications. Overall, the thickness of different types of oriented films spans from hundreds of nanometers to hundreds of micrometers. Such a substantial variation can lead to an order‐of‐magnitude difference in the output power of F‐TEDs. In addition to film thickness, the near‐room‐temperature *S* ^2^ *σ* of the film also plays a crucial role in determining the output performance of F‐TEDs [89, 90]. Figure 2c summarizes the near‐room‐temperature *S* ^2^ *σ* values of these highly oriented films, revealing significant variations not only among different types of oriented films but also within the same film category with different preferred crystallographic orientations [20, 43, 54, 59, 65, 67, 68, 69, 72, 73, 74, 75, 76, 77, 81, 84, 86, 91, 92].

## 3 Deposited Films

**FIGURE 3:**
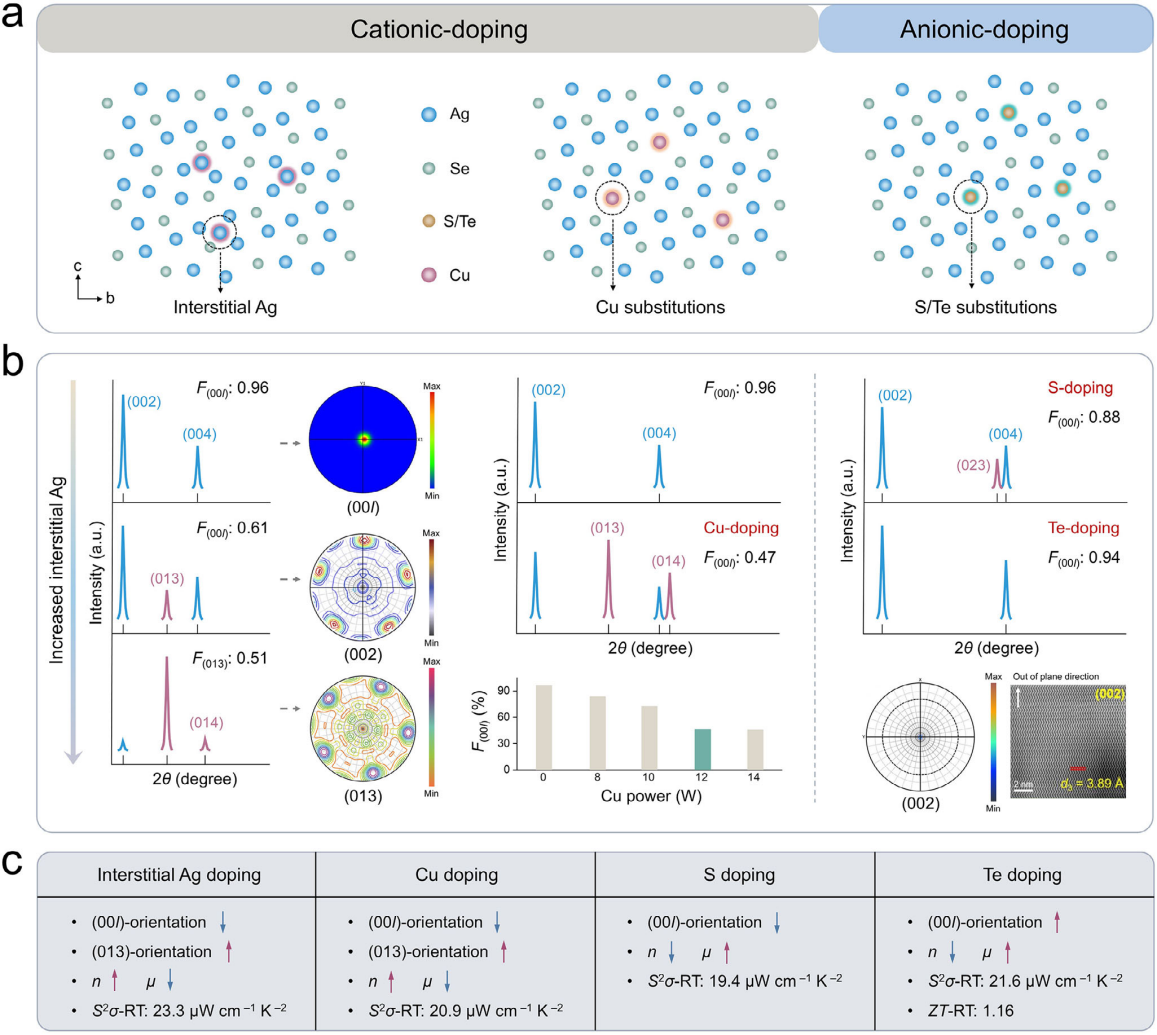
Overview of deposited Ag_2_Se films. (a) Schematic illustration of the atomic structures of deposited films containing different types of dopants. (b) Simplified X‐ray diffraction (XRD) patterns, pole figures, and orientation factor *F* of the deposited films. The inset transmission electron microscopy (TEM) image corresponds to the Te‐doped Ag_2_Se film, reproduced with permission [73]. Copyright 2024, Springer Nature. Pole files (top: reproduced with permission [67]. Copyright 2021, Elsevier; middle: reproduced with permission [68]. Copyright 2022, Royal Society of Chemistry; bottom: reproduced with permission [71]. Copyright 2024, Springer Nature; bottom right: reproduced with permission [73]. Copyright 2024, Springer Nature). (c) Summary of the effects of doping on crystal orientation, electrical transport properties (carrier concentration *n*, carrier mobility *μ*, room‐temperature power factor *S* ^2^ *σ*‐RT), and *ZT* of deposited films.

Deposited Ag_2_Se films are commonly prepared via VTE and MS methods, which enable precise control of the Ag/Se stoichiometric ratio and allow the introduction of extrinsic dopants, thereby modulating the preferred crystallographic orientation of the films. Such orientation tuning mainly involves the (00*l*) and (013) planes. Several theoretical studies have revealed pronounced anisotropy in the electrical transport properties of Ag_2_Se [14, 68, 76]. For instance, calculations of electrical transport along different crystallographic planes show that the band structure of the Ag_2_Se‐(002) plane exhibits semiconducting behavior, whereas the Ag_2_Se‐(013) plane displays semi‐metallic characteristics [68]. Consequently, Ag_2_Se films with a (002)‐preferred orientation tend to possess a lower carrier concentration (*n*) [68]. Cao et al. further calculated the charge density distribution on the (00*l*) and (013) planes of Ag_2_Se [76]. Their results indicate that the (00*l*) plane has a lower charge density than the (013) plane, resulting in a lower *n*. In addition, the (00*l*) plane exhibits a more ordered periodic atomic arrangement, which can suppress carrier scattering and thus enhance the *μ*. By summarizing recently published studies on deposited Ag_2_Se films, we find that cationic doping (e.g., interstitial Ag doping and Cu doping) generally enhances the (013) orientation while suppressing the (00*l*) orientation of the deposited films. In contrast, anionic doping (e.g., Te doping) tends to produce the opposite effect, as illustrated in Figure 3a,b.

Researchers have reported that deposited films with an Ag/Se ratio of approximately 1.99 exhibit a highly (00*l*)‐preferred orientation, attributed to the relatively low surface energy of the (00*l*) planes [67]. In this case, the orientation factor *F*_(00_ *_l_*_)_ can reach as high as 0.96. Introducing excess Ag (i.e., interstitial Ag) into the Ag_2_Se lattice (Ag/Se ratio > 2) suppresses the (00*l*) preferred orientation while enhancing the (013) orientation, thereby reducing *F*_(00_ *_l_*_)_ to 0.85. This phenomenon has been reported in subsequent studies [68, 71]. For example, Zheng et al. reported that a pronounced (00*l*) orientation can be obtained in ideally stoichiometric Ag_2_Se films. Increasing the Ag/Se ratio to 2.02 reduces *F*_(00_ *_l_*_)_ to 0.61 and simultaneously strengthens the (013) orientation of the films [68]. Further incorporation of interstitial Ag into the Ag_2_Se lattice can transform the (00*l*)‐preferred orientation into a (013)‐preferred orientation, leading to an *F*_(013)_ value of 0.51 for the Ag_2.19_Se film [71], as illustrated by the simplified X‐ray diffraction (XRD) patterns and pole figures in Figure 3b. The transformation in crystal orientation can be understood as follows. Excess Ag atoms in the Ag_2_Se lattice tend to occupy interstitial sites, which exhibit higher *μ* during the annealing process. This enhanced *μ* can locally destabilize the crystal lattice, facilitating recrystallization and consequently inducing a reorientation of the film [67]. In addition, theoretical calculations indicate that (013)‐oriented Ag_2_Se possesses a higher *n* than (00*l*)‐oriented Ag_2_Se, which is associated with the presence of interstitial Ag acting as electron donors [68, 76]. This strong coupling between composition and crystal orientation significantly influences the electrical transport performance of deposited films. Both the room‐temperature and peak *S* ^2^ *σ* values of the (013)‐oriented Ag_2.19_Se film are substantially enhanced compared with those of the (00*l*)‐oriented Ag_1.99_Se film, primarily due to the pronounced increases in *n* and *σ*, as shown in Figure 3c [67, 71].

Cu doping at Ag sites exerts a similar influence on crystal orientation to that of interstitial Ag doping. Researchers have employed a co‐sputtering strategy to introduce Cu into Ag_2_Se films, with the Cu doping concentration precisely controlled by adjusting the Cu sputtering power [54]. Cu incorporation in Ag_2_Se significantly suppresses the (00*l*) orientation while promoting the growth of the (013) and (014) planes, as illustrated by the simplified XRD patterns in Figure 3b [54]. With increasing Cu content, the intensities of the (002) and (004) diffraction peaks gradually decrease, and the *F*_(00_ *_l_*_)_ correspondingly declines from 0.96 for the undoped film to 0.47 for the Cu‐doped film prepared with a Cu sputtering power of 12 W [54]. This modification of crystal orientation also leads to an increase in *n* and an optimization of the *S* ^2^ *σ* near room temperature, as shown in Figure 3c [54]. It should be noted that deposited films are typically composed of nanocrystalline grains and therefore tend to exhibit relatively low *μ* and *σ*. Although adjusting the Ag/Se stoichiometric ratio or introducing Cu doping to promote the (013) orientation can effectively optimize *n* and *S* ^2^ *σ* near room temperature, these approaches inevitably lead to a reduction in *S*.

Unlike cationic doping, which generally enhances the (013) orientation, anionic doping (e.g., S and Te doping) exerts distinct effects on the crystal orientation of deposited Ag_2_Se films. For S doping, introducing a small amount of S atoms into the Ag_2_Se lattice can induce lattice distortion, thereby decreasing the *F*_(00_ *_l_*_)_ from 0.99 to 0.88, while additional diffraction peaks simultaneously appear in the XRD patterns, as shown in Figure 3b [72]. It should be noted that the influence of S doping on crystal orientation is relatively limited, and increasing the S‐doping concentration cannot further suppress the (00*l*) orientation [72]. Moreover, S doping into the Ag_2_Se lattice can reduce the concentration of interstitial Ag atoms and metastable phases, leading to a decrease in *n* and an increase in *μ*. In this case, the impact of S doping on electrical transport properties is more significant than that arising from the modulation of crystal orientation [72]. In contrast, Te doping exhibits a markedly different effect. Theoretical calculations reveal that substituting Te at Se sites can reduce the formation energy of the (00*l*) plane from 0.004 to 0.002 eV Å^−2^, thereby favoring the formation of highly (00*l*)‐oriented films [73]. The pristine Ag_2_Se film without Te doping displays a moderate (00*l*) preferred orientation with an *F*_(00_ *_l_*_)_ value of 0.61, accompanied by several other diffraction peaks. After Te doping, the growth of the (00*l*) plane is significantly promoted, resulting in a highly oriented film with an *F*_(00_ *_l_*_)_ value of 0.94 [73]. The pronounced (00*l*) orientation is further confirmed by pole figures and transmission electron microscopy (TEM) images of the Te‐doped Ag_2_Se film, as shown in Figure 3b. Te doping exerts a synergistic effect on the thermoelectric performance of Ag_2_Se films. From the perspective of electrical transport, the enhanced (00*l*) orientation leads to a decrease in *n* and an increase in *μ*, thereby optimizing the *S* ^2^ *σ* [73]. Meanwhile, in terms of thermal transport, Te doping introduces Te_Se_ point defects that effectively reduce *κ*_l_. As a result, the Ag_2_Se film doped with 3.2 at% Te exhibits a high room‐temperature *ZT* value of 1.16, as shown in Figure 3c [73].

## 4 Nanowire‐Based Films

**FIGURE 4:**
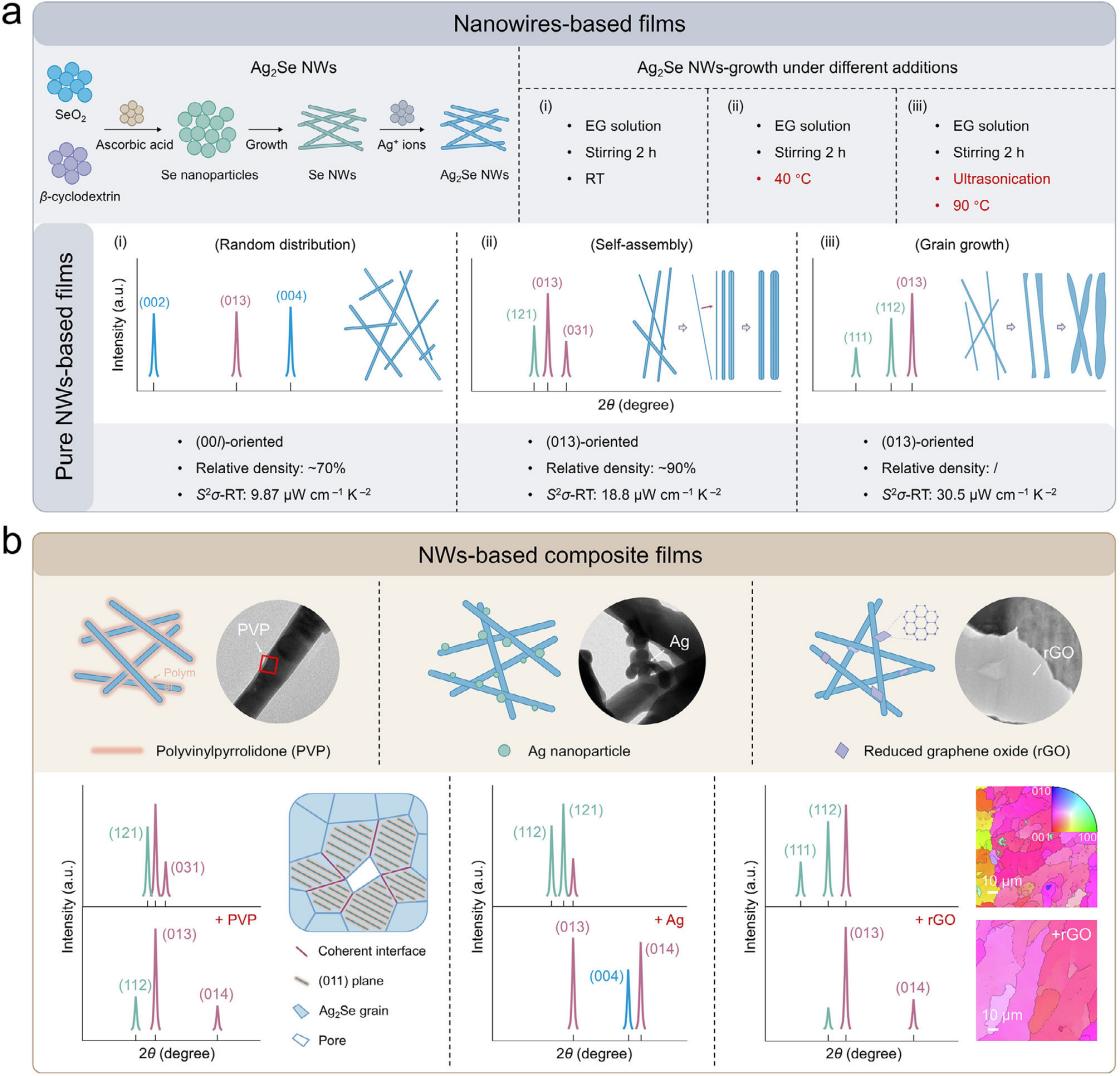
Overview of NWs‐based Ag_2_Se films. (a) Schematic illustration of the synthesis process of Ag_2_Se NWs and simplified XRD patterns of NWs‐based films prepared under different synthesis conditions, reproduced with permission [94]. Copyright 2019, Springer Nature. (b) Illustration of NWs‐based composite films, including representative TEM images (left: reproduced with permission [20]. Copyright 2021, Elsevier; middle: reproduced with permission [65]. Copyright 2021, American Chemical Society; right: reproduced with permission [77]. Copyright 2025, Springer Nature), simplified XRD patterns, and electron backscattered diffraction (EBSD) images, reproduced with permission [77]. Copyright 2025, Springer Nature.

In 2018, Ding et al. first reported a flexible Ag_2_Se NWs‐based film featuring a pronounced (00*l*) preferred orientation [14]. Benefiting from the high aspect ratio of the NWs and the flexibility of the nylon substrate, the fabricated Ag_2_Se film exhibited excellent mechanical flexibility [93]. However, two structural factors limited the *S* ^2^ *σ* of the film to relatively low values. First, network‐like NWs‐based films inevitably contain numerous submicron pores, which reduce the *μ* and *σ*. Second, the (00*l*) preferred orientation results in a lower *n* in Ag_2_Se, which is unfavorable for systems with intrinsically low *σ* [14, 20, 43, 65, 77]. To address these limitations, researchers have optimized the synthesis conditions to regulate the distribution and nanostructures of Ag_2_Se NWs, thereby modifying the preferred orientation and crystallinity of the resulting Ag_2_Se films, as illustrated in Figure 4a [14, 43, 77]. By simply adjusting the synthesis temperature of Ag_2_Se NWs, Jiang et al. successfully induced the self‐assembly of NWs and generated multi‐sized nanostructures, including thin NWs, thick NWs, and nanosheets [43]. These multi‐sized nanostructures facilitate the sintering process more effectively than NWs with uniform sizes. Consequently, a more compact Ag_2_Se film with an improved relative density of 90% was obtained, while the (00*l*) orientation was significantly suppressed, leading to a (013)‐preferred orientation in the Ag_2_Se film [43]. As a result, the synergistic enhancement of *μ* and *n* increased the room‐temperature *S* ^2^ *σ* from 9.87 to 18.8 µW cm^−1^ K^−2^ [43]. In subsequent work, Zhang et al. developed a high‐temperature‐assisted ultrasonication method to synthesize Ag_2_Se NWs with surface protrusions, which enabled the fabrication of (013)‐oriented Ag_2_Se films [77]. The combined effects of elevated temperature and ultrasonication promote the surface activity of amorphous Se and accelerate nucleation, leading to Se NWs with larger diameters and improved crystallinity. Moreover, the higher reaction temperature accelerates the formation of Ag_2_Se NWs and induces protrusions along the edges of the NWs [77]. These protrusions exhibit higher reactivity and increase the contact area between adjacent NWs, thereby enabling the formation of Ag_2_Se films with improved crystallinity, enhanced (013) orientation, and reduced porosity. Benefiting from the optimized crystal orientation and microstructure, the resulting Ag_2_Se film achieves an ultrahigh room‐temperature *S* ^2^ *σ* of 30.5 µW cm^−1^ K^−2^, as shown in Figure 4a [77].

Another effective strategy to modulate the crystal orientation of NWs‐based films is the composite approach, including in situ compositing with organic polymers, inorganic phases, and carbon‐based materials, as summarized in Figure 4b [20, 65, 77]. In the case of organic polymers, the organic coating layers can regulate crystal growth and induce coherent interfaces between adjacent grains, thereby promoting the (013) preferred orientation in the composite films. For example, Jiang et al. employed polyvinylpyrrolidone (PVP) as a binder to effectively restrain the anisotropic growth of Ag_2_Se grains and obtain a highly (013)‐oriented Ag_2_Se film [20]. In this system, PVP uniformly coats the surface of Ag_2_Se NWs and aligns the growth direction of neighboring grains during the film formation process, leading to coherent interfaces. In addition, PVP can function as a phonon‐scattering source, suppressing the *κ*_l_. As a result, the fabricated highly oriented Ag_2_Se film exhibits a high room‐temperature *S* ^2^ *σ* of ∼19.1 µW cm^−1^ K^−2^, corresponding to a *ZT* of ∼1.1 [20]. Inorganic compositing has also been demonstrated as an effective strategy. Gao et al. fabricated a series of Ag‐Ag_2_Se composite films by modulating the amount of Ag^+^ during the synthesis of Ag_2_Se NWs [65]. Ag nanoparticles were formed in situ alongside the growth of Ag_2_Se NWs. During the hot‐pressing process, molten Ag nanoparticles promote the sintering of NWs, thereby modifying the anisotropic characteristics of the Ag_2_Se film and enhancing the (013) and (014) preferred orientations of the composite films [65]. Apart from inorganic and organic additives, carbon‐based materials such as reduced graphene oxide (rGO) have also been introduced to regulate the microstructure and orientation of Ag_2_Se films [77]. The two‐dimensional rGO sheets can act as active nucleation sites to promote grain growth while simultaneously providing additional surface area to enhance grain contact and interaction. Figure 4b compares the simplified XRD patterns and electron backscattered diffraction (EBSD) images of Ag_2_Se films with and without rGO incorporation [77]. As observed, larger Ag_2_Se grains are obtained in the presence of rGO, and the (013) orientation of the composite films is significantly strengthened, resulting in enhanced *μ* and *σ*. Consequently, the fabricated Ag_2_Se‐rGO composite film achieves an outstanding room‐temperature *S* ^2^ *σ* of 37 µW cm^−1^ K^−2^ [77].

## 5 Selenized Films

Selenized films represent another class of highly oriented Ag_2_Se films, with thicknesses ranging from hundreds of nanometers to tens of micrometers [59, 69, 76, 84, 91, 92]. Unlike the previously discussed deposited films and NWs‐based films, whose crystal orientations are closely coupled with the elemental compositions and phase components of the films, the crystal orientation of selenized films is primarily governed by the film thickness and the selenization conditions.

**FIGURE 5:**
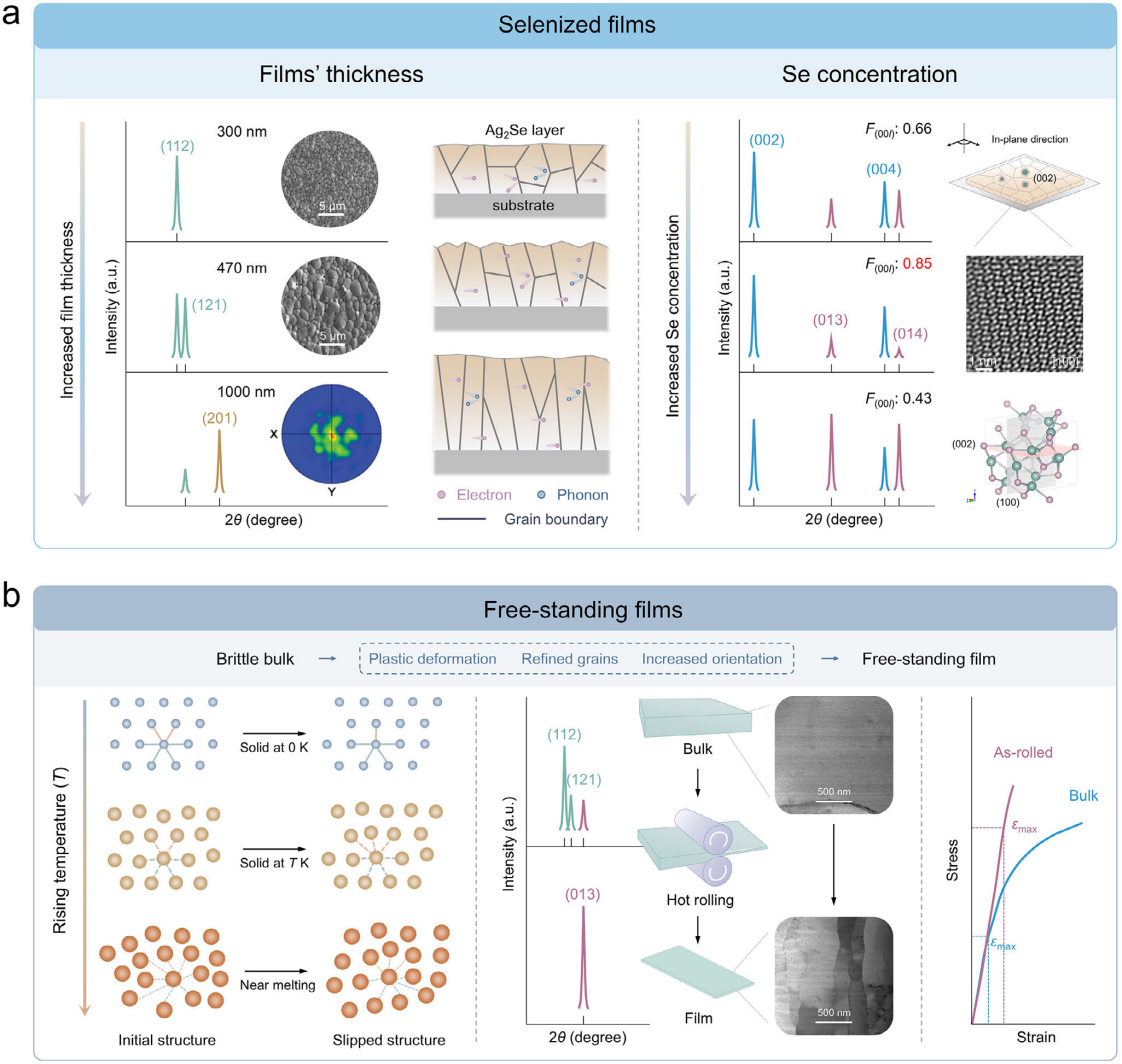
Selenized films and free‐standing Ag_2_Se films. (a) Structural characteristics of selenized Ag_2_Se films with out‐of‐plane and in‐plane preferred orientations, including a scanning electron microscopy (SEM) image: reproduced with permission [91]. Copyright 2025, American Chemical Society, TEM image: reproduced with permission [76]. Copyright 2025, Springer Nature, simplified XRD pattern, and pole figure: reproduced with permission [69]. Copyright 2022, Wiley. (b) Structural evolution of Ag_2_Se during plastic deformation. Schematic illustrations of atomic structures before and after slip at different temperatures, where brown lines represent broken and reconstructed bonds and cyan lines indicate intact bonds: reproduced with permission [86]. Copyright 2025, Springer Nature; the simplified XRD patterns and TEM images of the Ag_2_Se bulk and hot‐rolled film: reproduced with permission [86]. Copyright 2025, Springer Nature; the bending stress–strain curves of the Ag_2_Se bulk and hot‐rolled film: reproduced with permission [74]. Copyright 2024, Springer Nature. Here, maximum elastic strain is abbreviated as *ε*_max_.

The thickness of the fabricated Ag_2_Se films is typically controlled by adjusting the thickness of the precursor films prior to selenization. Researchers have employed MS to deposit elemental Ag on polyimide (PI) substrates, producing Ag precursor films with a (111) preferred orientation [69, 91]. Subsequent selenization reactions are carried out in Se‐containing aqueous solutions or vapor environments to convert the Ag precursor films into Ag_2_Se films. When the thickness of the Ag precursor film is approximately 300 nm, the resulting selenized film exhibits a pronounced (112) orientation [91]. As the thickness increases to 470 nm, the selenized film displays mixed (112) and (121) orientations [91]. When the precursor thickness increases to 1 µm, the preferred orientation of the selenized film transitions to the (201) plane [69]. Thicker precursor films generally require longer selenization times to achieve complete conversion. In addition, the in situ conversion of Ag to Ag_2_Se promotes grain growth along the direction perpendicular to the substrate, while grain growth parallel to the substrate is relatively suppressed [69]. Meanwhile, larger Ag_2_Se grains are formed with increasing precursor thickness. The simplified XRD patterns and corresponding pole figures confirm that the Ag_2_Se film exhibits a strong (201) preferred orientation, which is parallel to the in‐plane direction, as shown in Figure 5a [69]. The selenized films typically exhibit a columnar morphology with random grain distribution in the in‐plane direction and a pronounced (201) out‐of‐plane orientation. Such a structural feature is advantageous for decoupling electron and phonon transport [69]. The randomly distributed grain boundaries along the in‐plane direction effectively scatter phonons, while the highly oriented columnar grains maintain efficient electronic transport [69]. As a result, the Ag_2_Se film achieves a high *S* ^2^ *σ* of ∼26 µW cm^−1^ K^−2^ and a low *κ* of ∼0.66 W m^−1^ K^−1^ at room temperature, leading to a room‐temperature *ZT* of ∼1.2 [69]. In subsequent studies, Wu et al. replaced the (111)‐oriented Ag precursor film with a (013)‐oriented Ag_2_(S, Se) plastic film as the substrate for the selenization reaction [84, 92]. Oriented Ag_2_Se films grow on the surface of the Ag_2_(S, Se) film, and the Ag_2_Se grains progressively elongate along the out‐of‐plane direction with increasing selenization time, exhibiting a similar thickness‐dependent evolution of crystal orientation [84]. More importantly, the fabricated Ag_2_Se films possess thicknesses on the order of tens of micrometers and exhibit a high average *S* ^2^ *σ* of ∼27 µW cm^−1^ K^−2^ in the temperature range of 298–393 K, demonstrating promising potential for practical applications [92].

Modulating the Se concentration in aqueous solutions provides an effective route to regulate the crystal orientation of selenized Ag_2_Se films. Researchers proposed a wet‐chemical selenization strategy in which the Se concentration is tuned to control the in‐plane orientation of the resulting films, thereby suppressing the growth of the (013) and (014) planes while maximizing the (00*l*) orientation [76]. Figure 5a compares the simplified XRD patterns and the corresponding *F*_(00_ *_l_*_)_ of Ag_2_Se films selenized in aqueous solutions with different Se concentrations. The results show that *F*_(00_ *_l_*_)_ first increases and then decreases with increasing Se concentration, indicating the presence of an optimal Se concentration for achieving highly (00*l*)‐oriented Ag_2_Se films [76]. Unlike the previously discussed out‐of‐plane oriented Ag_2_Se films, these films exhibit a pronounced in‐plane preferred orientation. High‐resolution TEM (HRTEM) images of the cross‐sectional morphology reveal a clear lattice arrangement corresponding to the [100] axis of orthorhombic Ag_2_Se, indicating that the in‐plane direction is parallel to the (00*l*) plane, which is consistent with the XRD results [76]. The significantly enhanced (00*l*) orientation enables an ultrahigh *μ* of 1500 cm^2^ V^−1^ s^−1^, resulting in an *S* ^2^ *σ* of ∼31 µW cm^−1^ K^−2^ at 343 K [76].

## 6 Free‐Standing Films

Unlike plastic semiconductors such as Ag_2_S, InSe, and Mg_3_Bi_2_, which exhibit intrinsic plastic deformability, Ag_2_Se is generally brittle and can sustain only limited strain under compressive and bending conditions (compressive strain: ∼12%; bending strain: ∼5%) [74, 95]. Recently, however, researchers have found that Ag_2_Se can undergo plastic deformation at moderately elevated temperatures, particularly near its phase transition point [74, 75, 81, 86]. This temperature‐dependent plasticity originates from thermally activated lattice deformation [86]. At 0 K, atoms are rigidly bonded to their neighboring atoms and remain essentially immobile. As the temperature increases, atomic vibrations intensify, facilitating the movement of lattice planes and defects and thereby introducing a certain degree of plasticity. When the temperature approaches the melting point of the material, the strongly vibrating atoms can partially flow, resulting in significantly enhanced plastic deformability, as illustrated in Figure 5b [86]. Consequently, the intrinsically brittle Ag_2_Se ingot can be hot‐rolled into free‐standing thin films through a simple warm‐metalworking process.

Microstructural characterizations reveal that plastic deformation can significantly refine and elongate Ag_2_Se grains, resulting in a pronounced (013) preferred orientation and distinctive microstructural features in the free‐standing films [81]. After hot rolling, large Ag_2_Se grains tend to fragment into smaller grains, forming intensive low‐angle grain boundaries between adjacent grains [81]. Meanwhile, the applied extrusion pressure elongates the grains along the rolling direction, leading to increased grain length along this direction and consequently inducing a strong (013) preferred orientation in the rolled film, as illustrated in Figure 5b [81]. The resulting (013)‐oriented Ag_2_Se film exhibits anisotropic electrical transport behavior and possesses a higher *n* than the unrolled bulk material. The room‐temperature *S* ^2^ *σ* along the in‐plane direction (21 µW cm^−1^ K^−2^) is significantly higher than that along the out‐of‐plane direction (13 µW cm^−1^ K^−2^). This anisotropic transport behavior originates from the different degrees of carrier scattering along the two directions. Specifically, the elongated grains introduce a higher intensity of grain boundaries along the out‐of‐plane direction, which enhances carrier scattering and thereby reduces the electrical transport performance in that direction [81].

In addition to the anisotropic electrical transport properties, researchers have also found that the hot‐rolling process can significantly enhance the flexibility of Ag_2_Se films [74]. A higher elastic strain allows the film to undergo safe and recoverable bending at smaller radii for a given thickness. The as‐rolled film exhibits a remarkable maximum elastic strain ($\varepsilon_{E}^{max}$) of ∼0.8%, which is approximately twice that of the unrolled ingot (Figure 5b) [74]. As a result, a 36 µm thick film maintains nearly unchanged electrical transport performance after undergoing 1 000 000 elastic bending cycles at a bending radius of ∼3 mm [74]. Such outstanding flexibility surpasses that of most previously reported flexible Ag_2_Se‐based films [74]. Furthermore, researchers have demonstrated that sulfur alloying in Ag_2_Se can effectively reduce the hot‐rolling temperature to near room temperature [75]. In general, plastic deformation at moderately elevated temperatures enables the fabrication of highly oriented free‐standing Ag_2_Se films [75]. The resulting elongated grains exhibit a pronounced (013) preferred orientation, ensuring a high *n* in the rolled films. Meanwhile, the refined grains combined with the presence of dislocations contribute to improved mechanical flexibility [75].

## 7 Conclusions

Owing to their excellent near‐room‐temperature thermoelectric performance and considerable mechanical flexibility across a wide range of thicknesses, highly oriented Ag_2_Se films and their corresponding devices exhibit great potential for applications in self‐powered wearable systems [96]. Over the past five years, remarkable progress has been achieved in highly oriented Ag_2_Se films, spanning fundamental understanding of orientation mechanisms, performance optimization, and device‐level demonstrations. For instance, an ultrahigh room‐temperature *S* ^2^ *σ* of 37 µW cm^−1^ K^−2^ has been achieved in rGO‐composited Ag_2_Se films with a (013) preferred orientation, and the assembled F‐TED can power a thermo‐hygrometer and a wristwatch, demonstrating significant practical potential. In addition, a selenized Ag_2_Se film with an out‐of‐plane preferred orientation has achieved an outstanding room‐temperature *ZT* of 1.2, while a four‐leg F‐TED fabricated from this film delivers a high *ω* of 124 W cm^−2^. Despite these rapid advances, substantial challenges remain regarding the fundamental mechanisms governing crystal‐orientation evolution as well as the trade‐off between thermoelectric performance and mechanical flexibility. In the following section, we summarize the key challenges and outline future research directions for different types of highly oriented Ag_2_Se films.

### 7.1 Deposited Films

Among the four types of highly oriented films, deposited films typically exhibit the smallest thickness (hundreds of nanometers) and therefore possess excellent mechanical flexibility. To ensure sufficient output power in the assembled lateral devices, deposited films must exhibit superior electrical transport properties. However, the grain size of deposited films usually ranges from only several hundred nanometers, resulting in a high intensity of grain boundaries that significantly suppresses the *μ*. Currently, the primary strategy for improving the *S* ^2^ *σ* is to promote the (013) preferred orientation to increase the *n*. However, this approach inevitably leads to a reduction in the *S*, resulting in lower *S* ^2^ *σ* values for deposited films compared with other types of oriented films. Developing novel deposition techniques, such as chemical bath deposition or electrochemical deposition, to fabricate (00*l*)‐oriented Ag_2_Se films composed of larger microcrystals could provide an effective solution. In combination with rational post‐treatments (e.g., annealing or hot pressing) to induce recrystallization, the grain boundary intensity may be reduced, thereby enhancing *μ* and ultimately improving *S* ^2^ *σ*. In addition, introducing suitable secondary phases or implementing in situ compositing during the deposition process to establish highly conductive channels represents another promising strategy for enhancing *μ*. These secondary phases may also function as “binders” that strengthen the interfacial bonding between adjacent grains, thereby further improving the mechanical flexibility of the films. Furthermore, although current studies have revealed correlations between elemental composition and the preferred orientation of deposited films, the underlying mechanisms governing these phenomena remain insufficiently understood. Future investigations combining in situ characterization of the deposition process with advanced computational modeling are expected to provide deeper insights into the orientation evolution mechanism and guide the rational design of high‐performance deposited Ag_2_Se films. Besides, the current doping‐induced orientation engineering only involves Cu, S, and Te elements. Further investigations should focus on developing new and effective dopants, such as Sn, AI, and Zn elements, which have been demonstrated as effective dopants in Ag_2_Se‐based bulks.

### 7.2 NWs‐Based Films

The high aspect ratio of Ag_2_Se NWs not only endows the resulting films with excellent mechanical flexibility, even at thicknesses of tens of micrometers, but also facilitates the formation of a (013) preferred orientation. Modulating the morphology of NWs and introducing secondary phases have both proven effective in promoting the (013) orientation in NWs‐based films, leading to excellent thermoelectric performance near room temperature. However, in‐depth investigations into the formation mechanism of such oriented films remain limited. In particular, the structural evolution of NWs during the film formation process plays a critical role in understanding the origin of the (013) orientation. A more comprehensive and mechanistic understanding of this process would not only clarify the orientation formation pathway but also guide the fabrication of NWs‐based films with alternative preferred orientations, such as (00l) and (201), which are expected to exhibit superior TE performance compared with (013)‐oriented films. Furthermore, future research should focus on elucidating the specific role of secondary phases in regulating the crystal orientation of NWs‐based films. Systematic exploration of suitable secondary phases such as low‐dimensional materials, conductive polymers, and functional nanoparticles may provide new opportunities for tailoring the microstructure, enhancing carrier transport, and further improving the thermoelectric performance of NWs‐based Ag_2_Se films. More importantly, synthesizing Ag_2_Se nanorods or nanosheets with larger grain sizes may be beneficial for achieving highly oriented Ag_2_Se films with improved thermoelectric performance.

### 7.3 Selenized Films

Selenized films typically exhibit an out‐of‐plane preferred orientation, and their columnar microstructures are beneficial for decoupling electrical and thermal transport, thereby enabling excellent thermoelectric performance near room temperature. However, such a microstructure is unfavorable for flexible deformation. During bending, cracks tend to initiate and propagate along grain boundaries perpendicular to the in‐plane direction of the film, leading to the deterioration of electrical transport properties. This adverse effect becomes more pronounced in thicker films. Therefore, developing effective strategies to mitigate the trade‐off between thermoelectric performance and mechanical flexibility in selenized films is urgently required. Introducing suitable nano‐binders between columnar grains during the selenization process may provide an effective approach to enhance flexibility while simultaneously generating additional interfacial effects that could further optimize TE performance. Moreover, rational post‐treatments to modulate the interfacial structures, as well as the design of precursor films with tailored geometries, may offer promising routes to regulate the mechanical properties of selenized films while maintaining their high thermoelectric performance.

### 7.4 Free‐Standing Films

The rapid development of plastic semiconductors and plastic‐processing technologies has enabled the fabrication of free‐standing Ag_2_Se films with a (013) preferred orientation. These films can maintain considerable mechanical flexibility even at thicknesses of hundreds of micrometers, demonstrating promising potential for practical applications. However, their electrical transport performance remains relatively limited, which can be attributed to the high intensity of grain boundaries and dislocations introduced during the plastic deformation process. Although these lattice imperfections enhance mechanical flexibility, they also increase electron scattering and consequently deteriorate the electrical transport properties of the free‐standing films. Developing rational post‐treatment strategies therefore represents a promising approach to regulate the intensity of lattice imperfections, thereby balancing mechanical flexibility and electrical transport performance in free‐standing films.

## Author Contributions

**Qingfeng Liu**: conceptualization, supervision, formal analysis, project administration, resources, investigation, funding acquisition, Writing – review and editing. **Xiao‐Lei Shi**: conceptualization, data curation, supervision, resources, project administration, formal analysis, validation, visualization, investigation, writing – review and editing. **Zhi‐Gang Chen**: conceptualization, data curation, supervision, project administration, resources, formal analysis, validation, investigation, writing – review and editing. **Hao Wu**: software, data curation, formal analysis, validation, visualization, writing – original draft.

## Conflicts of Interest

The authors declare no conflicts of interest.

## Data Availability

Data sharing not applicable to this article as no datasets were generated or analysed during the current study.
